# Novel self-epitopes derived from aggrecan, fibrillin, and matrix metalloproteinase-3 drive distinct autoreactive T-cell responses in juvenile idiopathic arthritis and in health

**DOI:** 10.1186/ar2088

**Published:** 2006-11-27

**Authors:** Sylvia Kamphuis, Kolbrún Hrafnkelsdóttir, Mark R Klein, Wilco de Jager, Margje H Haverkamp, Jolanda HM van Bilsen, Salvatore Albani, Wietse Kuis, Marca HM Wauben, Berent J Prakken

**Affiliations:** 1 Department of Paediatric Immunology and IACOPO, Institute for Translational Medicine, University Medical Center Utrecht, PO Box 85090, 3508 AB Utrecht, The Netherlands; 2 Department of Paediatric Immunology and Rheumatology, Erasmus MC Sophia, PO Box 2060, 3000 CB Rotterdam, The Netherlands; 3 Department of Infectious Diseases and Immunology, Faculty of Veterinary Medicine, Utrecht University, Yalelaan 1, 3584 CL Utrecht, The Netherlands; 4 Department of Medicine and Pediatrics and IACOPO Institute for Translational Medicine, University of California San Diego, 9500 Gilmandrive, La Jolla CA 92093-0663, USA; 5 Androclus Therapeutics, Via Carducci 15, 92100 Milan, Italy; 6 Department of Biochemistry and Cell Biology, Faculty of Veterinary Medicine, Utrecht University, Yalelaan 1, 3584 CL Utrecht, The Netherlands

## Abstract

Juvenile idiopathic arthritis (JIA) is a heterogeneous autoimmune disease characterized by chronic joint inflammation. Knowing which antigens drive the autoreactive T-cell response in JIA is crucial for the understanding of disease pathogenesis and additionally may provide targets for antigen-specific immune therapy. In this study, we tested 9 self-peptides derived from joint-related autoantigens for T-cell recognition (T-cell proliferative responses and cytokine production) in 36 JIA patients and 15 healthy controls. Positive T-cell proliferative responses (stimulation index ≥2) to one or more peptides were detected in peripheral blood mononuclear cells (PBMC) of 69% of JIA patients irrespective of major histocompatibility complex (MHC) genotype. The peptides derived from aggrecan, fibrillin, and matrix metalloproteinase (MMP)-3 yielded the highest frequency of T-cell proliferative responses in JIA patients. In both the oligoarticular and polyarticular subtypes of JIA, the aggrecan peptide induced T-cell proliferative responses that were inversely related with disease duration. The fibrillin peptide, to our knowledge, is the first identified autoantigen that is primarily recognized in polyarticular JIA patients. Finally, the epitope derived from MMP-3 elicited immune responses in both subtypes of JIA and in healthy controls. Cytokine production in short-term peptide-specific T-cell lines revealed production of interferon-γ (aggrecan/MMP-3) and interleukin (IL)-17 (aggrecan) and inhibition of IL-10 production (aggrecan). Here, we have identified a triplet of self-epitopes, each with distinct patterns of T-cell recognition in JIA patients. Additional experiments need to be performed to explore their qualities and role in disease pathogenesis in further detail.

## Introduction

Juvenile idiopathic arthritis (JIA) is a heterogeneous autoimmune disease of childhood and is characterized by chronic inflammation of one or more joints [1,2]. Inflammation in the joint is characterized by a selected accumulation in the synovium of activated T cells, which are clustered around antigen-presenting (dendritic) cells [3,4]. Oligoclonal expansions of T cells are present in synovial fluid as compared with peripheral blood and these T cells show an activated, highly differentiated memory phenotype that implies selective recruitment from the blood [5,6]. In addition, JIA has strong associations with multiple human leukocyte antigen (HLA) genes, class II associations being more numerous than the few documented class I associations [1,7-10]. Altogether, this supports the concept of an ongoing antigen-driven immune response with a central role for autoreactive T cells recognizing antigens that are expressed in the joint in the context of HLA.

Knowing which antigens drive or control autoreactive T-cell responses in JIA is important for our understanding of the role of T cells in disease pathogenesis. In addition, identification of such self-epitopes may provide us with tools to monitor JIA-specific T-cell responses during the course of the disease, and may become targets for antigen-specific immune therapy. At present, only a limited number of self-antigens involved in JIA disease pathogenesis are known [11-15]. A popular and attractive hypothesis on how autoreactive T cells can trigger autoimmune pathology is the 'molecular mimicry' scenario: activation of autoreactive lymphocytes by structurally similar antigenic determinants of infectious pathogens [16,17]. Molecular mimicry at the level of CD4^+ ^T cells is suggested to be involved in several autoimmune diseases, including JIA [14,18-24], although definite proof for such a mechanism is lacking in most cases [17].

The aim of the present study was to identify joint-related self-antigens that induce T-cell reactivity in patients with JIA. The self-epitopes tested in this study were recently identified as T-cell epitopes recognized during adjuvant arthritis (AA) [25]. AA is an experimental rat model for chronic arthritis with resemblance to JIA and rheumatoid arthritis (RA) [26]. The novel T-cell epitopes were selected with an elaborate computer search strategy based on the alleged molecular mimicry scenario as a cause for disease induction in AA [25]. Previously, a selection of human homologs of these epitopes were tested for T-cell recognition in patients with RA [27]. Based on the T-cell responses noted in AA and RA, we selected a set of nine human homologs of the identified self-epitopes and tested them for T-cell recognition in patients with JIA. All epitopes were conserved, differing by at most three amino acids between the rat and human sequences.

We found that self-epitopes derived from aggrecan, fibrillin, and matrix metalloproteinase (MMP)-3 induced in patients with JIA significant T-cell responses that were related to disease duration and disease subtype. In contrast to the epitopes derived from aggrecan and fibrillin, the MMP-3-derived epitope was also recognized in healthy controls. Restimulated peptide-specific T-cell lines of patients with polyarticular JIA showed production of interferon-γ (IFN-γ)/interleukin (IL)-17 and inhibition of IL-10 production. Here, we have identified a triplet of self-epitopes derived from joint-related structures and each one shows a distinct profile of T-cell recognition in JIA and in health.

## Materials and methods

### Subjects

We obtained blood from 36 JIA patients who fulfilled the diagnostic criteria of oligoarticular and polyarticular (including extended oligoarticular) onset types [28]. Patient characteristics are presented in Table 1. We obtained blood from 15 healthy children (age range 3.0 to 12.6 years, median 7.0 years; 7 male, 8 female), who were undergoing minor surgical procedures, as controls. Informed consent was obtained from all children or their parents. The local medical ethics review board approved the study.

### Peptide selection and synthesis

The joint-related self-epitopes were selected on the basis of the molecular mimicry computer search described by van Bilsen and colleagues [25]. Briefly, the interaction of the mycobacterial heat-shock protein 65 (HSP65) 178–186 peptide with rat major histocompatibility complex (MHC) class II (RTl.B^L^) and the T-cell receptor (TCR) of an arthritogenic T-cell clone was used as a mold for the identification of fitting self-epitopes. Because it is still debated whether T-cell epitopes derived from autoantigens display high or low affinity for their MHC restriction element, the search profile was designed to select peptides with a broad range of affinities to RTl.B^L ^[29-31]. Based on the T-cell responses noted in AA and RA [27,32], we selected a set of nine human homologs of the identified self-epitopes and tested them for T-cell recognition in patients with JIA. Peptide characteristics are shown in Table 2.

The peptides were synthesized as 15-mers via automated simultaneous multiple peptide synthesis (SMPS). The SMPS set-up was developed using a standard auto sampler (Gilson 221) as described previously [33]. Briefly, standard Fmoc chemistry with *in situ *PyBop/NMM activation of the amino acids in fivefold molar excess with respect to 2 μmol/peptide PAL-PEG-PS resin (PerSeptive Biosystems, now part of Applied Biosystems, Foster City, CA, USA) was employed. Peptides were obtained as C-terminal amides after cleavage with 90%–95% TFA/scavenger cocktails. Peptides were analyzed and purified by reverse-phase high performance liquid chromatography (HPLC) and checked via electrospray ionization mass spectrometry (LCQ; Thermoquest, now part of Thermo Electron Corporation, Waltham, MA, USA). If peptide purity was less than 95%, peptides were purified on reverse-phase HPLC before use.

### T-cell proliferation assays

Peripheral blood mononuclear cells (PBMC) were isolated by Ficoll Paque (Amersham Pharmacia Biotech, now part of GE Healthcare, Little Chalfont, Buckinghamshire, UK) density gradient centrifugation of heparinized blood. Cells were washed and cultured in RPMI-1640 supplemented with 2 mM glutamine, 100 U/ml of penicillin/streptomycin (Gibco BRL, now part of Invitrogen Corporation, Carlsbad, CA, USA), and 10% (by volume) AB^pos ^heat-inactivated (60 minutes at 56°C) human serum (Sanquin Blood Bank, Utrecht, The Netherlands). Cells were cultured in triplicate (2 × 10^5 ^cells in 200 μl per well) in round-bottomed 96-well plates (NUNC A/S, Roskilde, Denmark) for 120 hours at 37°C in 5% CO_2 _with 100% relative humidity, in the absence or presence of 20 μg/ml peptide. These concentrations were found to be optimal in preliminary dose-response experiments (data not shown). Concanavalin A (2.5 μg/ml; Calbiochem, San Diego, CA, USA) and tetanus-toxoid (1.5 μg/ml; RIVM, Bilthoven, The Netherlands) were used as positive controls. A mouse class II restricted epitope (OVA 323–339) was used as an irrelevant control peptide. During the last 16 hours of culture, 1 μCi (37 kBq) ^3^H-thymidine (ICN Biomedicals, now part of MP Biomedicals, Irvine, CA, USA) was added to each well. Cells were harvested according to standard procedures, and incorporated radioactivity was measured by liquid scintillation counting and expressed as counts per minute (cpm). The magnitude of the proliferative response was expressed as stimulation index (SI), which is calculated as the mean cpm of cells cultured with antigen divided by the mean cpm of cells cultured without antigen.

### Analysis of peptide-induced cytokine production by multiplexed particle-based flow cytometric assay

T-cell cultures were performed as described above with PBMC of 24 patients with JIA and 12 healthy controls. After 96 hours, cell culture supernatants were collected and stored at -80°C. Cytokine concentrations were measured with the Bio-Plex system in combination with the Bio-Plex Manager software, version 3.0 (Bio-Rad Laboratories, Inc., Hercules, CA, USA), which employs the Luminex xMAP technology as previously described [34-37]. Briefly, this technology uses pairs of antibodies directed against non-competing epitopes of their respective analytes. One of the antibodies is covalently bound to a fluorescence-emitting microsphere (λ_1 _and λ_2_) and the other biotinylated antibody is bound to streptavidin-phycoerythrin (PE) (λ_3_). The two-dimensional signal (λ_1 _and λ_2_) discriminates between up to 100 different microspheres in a single sample. The second signal (λ_3_) quantifies the exact amount of a particular microsphere in a sample. Fluorescent intensity of the microsphere complex was measured in a final volume of 100 μl of high performance enzyme-linked immunosorbent assay buffer. The antibody pairs were purchased and coupled as previously described [35,37]. Peptide-specific cytokine production is calculated as the cytokine production of cells cultured with peptide subtracted by the cytokine production of cells cultured without peptide. The following cytokines were measured: IL-1α, IL-1β, IL-5, IL-10, IL-12, IL-18, tumor necrosis factor-α (TNF-α), and IFN-γ.

### Cytokine analysis by lymphocyte intracellular staining and flow cytometry

T-cell cultures were performed as described above for 72 hours with 4 × 10^5 ^cells in 200 μl per well using PBMC from 14 patients with JIA and 6 healthy controls. During the last 5 hours of culture, Golgistop (BD Biosciences, San Jose, CA, USA) was added (2 μM final concentration). The cells were harvested, washed in cold phosphate-buffered saline (PBS) with 2% fetal calf serum (FCS), blocked in PBS with 10% FCS for 20 minutes at 4°C, washed twice, and stained with CyChrome-conjugated anti-CD4 for 20 minutes at 4°C. Subsequently, the cells were fixed in Cytofix/Cytoperm solution (BD Biosciences) for 20 minutes at 4°C and washed twice in Perm/Wash solution (BD Biosciences). After the second wash, the cells were resuspended in 50 μl of Perm/Wash solution containing a predetermined optimal concentration of PE-conjugated anti-IL-10, PE-conjugated anti-IL-4, fluorescein isothiocyanate (FITC)-conjugated anti-IFN-γ, and FITC-conjugated anti-TNF-α. After incubation for 30 minutes at 4°C, cells were washed twice. Stained mononuclear cells were diluted in sheath fluid and run on a FACSCalibur flow cytometer (BD Biosciences). CellQuest software (BD Biosciences) was used for analysis.

### Short-term T-cell lines

PBMC from five patients with polyarticular JIA were cultured in RPMI-1640 supplemented with 2 mM glutamine, 100 U/ml of penicillin/streptomycin (Gibco BRL, now part of Invitrogen Corporation), and 10% AB^pos ^heat-inactivated (60 minutes at 56°C) human serum (Sanquin Blood Bank) at 2 × 10^5 ^cells per well in the presence of 20 μg/ml peptide (aggrecan, MMP-3, fibrillin). On days 4, 8, and 12, the culture medium was refreshed and IL-2 (40 U/ml; Roche, Almere, The Netherlands) was added. The cells were restimulated with peptide (20 μg/ml) on day 10. After a total of 14 days of culture, the cells were harvested and stained with CD3 (clone SK3), IFN-γ (clone 4S.B3), and IL-10 (clone JES3-19F1; all BD Biosciences) for fluorescence-activated cell sorting analysis as described above. In addition, culture supernatants were collected and analyzed with multiplexed particle-based flow cytometric assay (as described above) for production of cytokines (IL-1α, IL-1β, IL-4, IL-10, IL-17, TNF-α, IFN-γ, and CXCL8).

### HLA typing

Eleven patients with JIA (three oligoarticular JIA, eight polyarticular JIA) were HLA-typed for HLA class II antigens DRB1, DRB3-5, and DQB1 by PCR-SSP (polymerase chain reaction-sequence-specific primer) according to the manufacturer's protocol (GenoVision, Vienna, Austria).

### Statistical analysis

Statistical evaluation was performed using SPSS software, version 12.0 (SPSS Inc., Chicago, IL, USA). Basic descriptive statistics were used to describe the patient population. Group differences were analyzed with the Mann-Whitney *U *test. Group differences were adjusted for possible confounders (age and gender) using linear regression with a group indicator. A *p *value of less than 0.05 was considered statistically significant.

## Results

### T-cell recognition of aggrecan, fibrillin, and MMP-3

**Figure 1:**
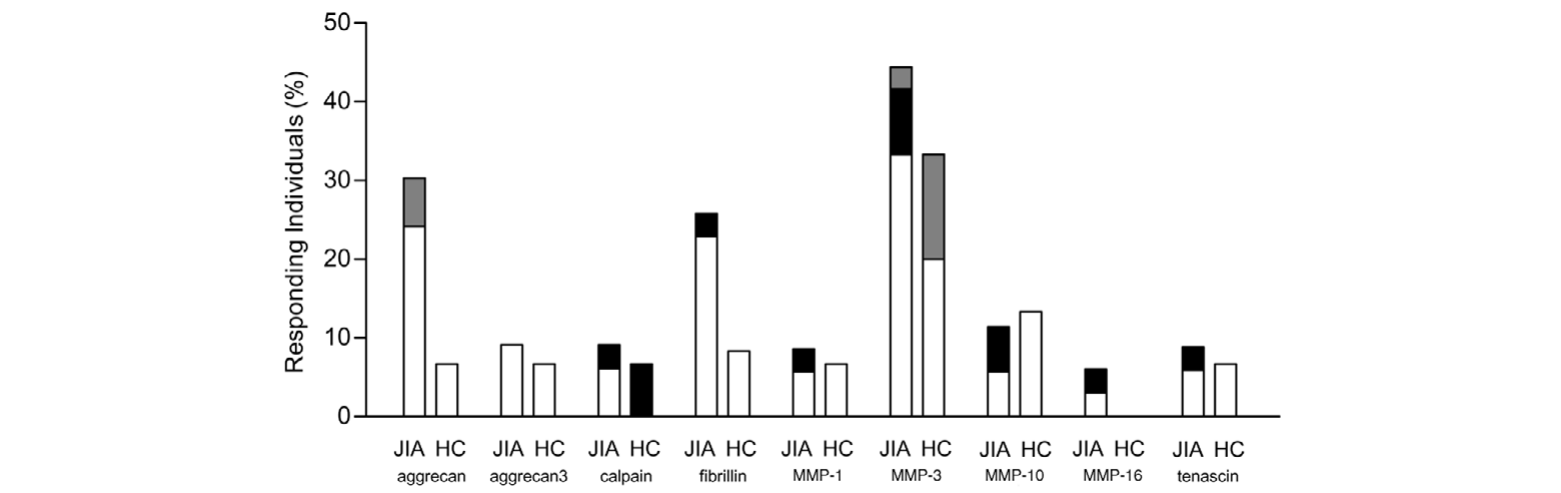
Percentage of patients with juvenile idiopathic arthritis (JIA) and healthy controls showing positive T-cell proliferative responses. Transparent bar: 2 ≤ stimulation index (SI) < 3; black bar: 3 ≤ SI < 4; gray bar: SI ≥ 4. The highest frequencies were found for the peptides derived from aggrecan, fibrillin, and matrix metalloproteinase (MMP)-3.

**Figure 2:**
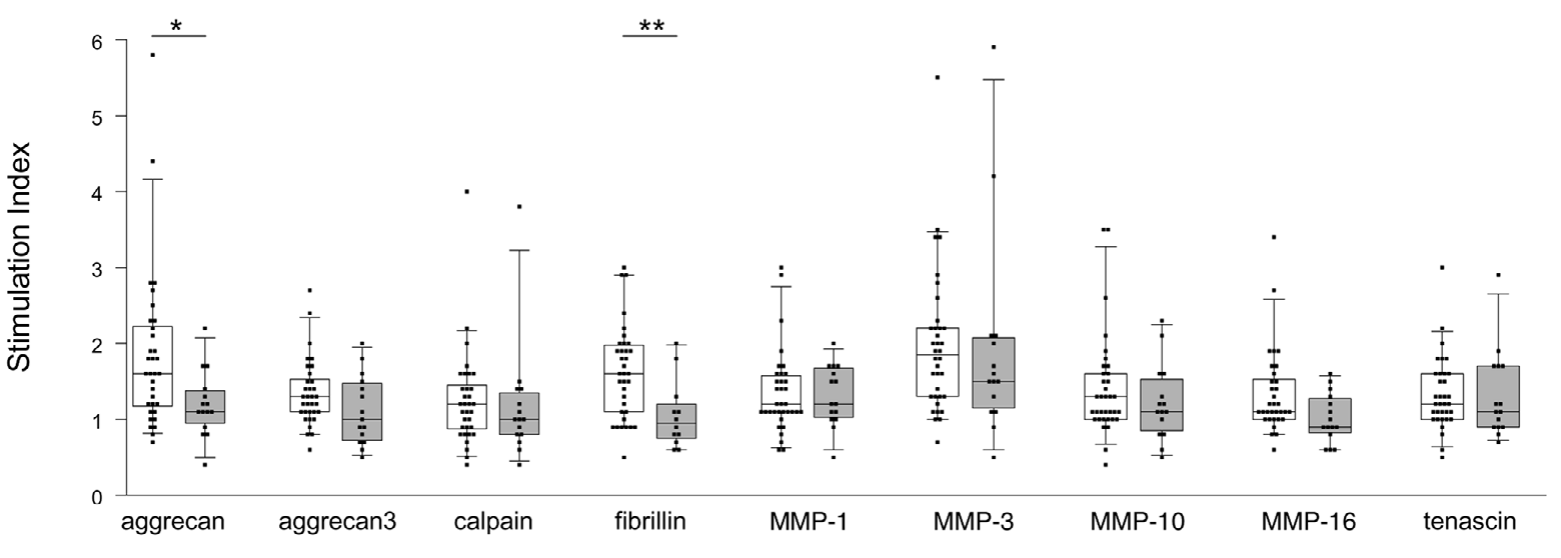
T-cell proliferative responses in patients with juvenile idiopathic arthritis (JIA) and healthy controls. Proliferative responses are presented as interquartile range for each group of data, with horizontal lines showing the median. The aggrecan and fibrillin peptides induced significant differences in responses of peripheral blood mononuclear cells (PBMC) from patients with JIA and healthy controls and are indicated as follows: **p = 0.005, *p = 0.015. The matrix metalloproteinase (MMP)-3 peptide induced proliferative responses in PBMC of patients with JIA as well as in PBMC of controls. Transparent boxes: JIA; gray boxes: healthy controls.

Positive T-cell proliferative responses (SI ≥ 2) to one or more peptides were detected in PBMC from 25 of 36 patients with JIA (69%). The peptides derived from aggrecan, fibrillin, and MMP-3 yielded the highest frequency of T-cell proliferative responses in patients with JIA (Figure 1). Proliferative responses to the aggrecan and fibrillin peptides were significantly different from those induced in PBMC from healthy controls (aggrecan, *p *= 0.015; fibrillin, *p *= 0.005) (Figure 2). Adjustment for age and gender did not explain these differences. Positive proliferative responses to the MMP-3 peptide were detected in PBMC from both patients with JIA and healthy controls.

Further analysis of the 25 JIA patients with positive T-cell responses to one or more peptides revealed that 11 JIA patients (44%) responded to 1 of 9 peptides, 3 JIA patients (12%) responded to 2 of 9 peptides, 8 JIA patients (32%) responded to 3 of 9 peptides, and 3 JIA patients (12%) responded to 4 of 9 peptides. All JIA patients with positive T-cell responses to two or more peptides responded to variable peptide combinations (data not shown). In addition, we did not note any correlation between JIA patients with positive T-cell responses to two or more peptides and disease subtype or disease duration. Two patients with rheumatoid factor-positive JIA were included in the study; one patient did not respond to any of the tested peptides, and the other responded to three peptides (aggrecan, calpain, and MMP-16).

Patients with JIA are known to have polymorphic MHC genotypes [1], which was confirmed via HLA-class II genotyping of selected JIA in our study. In addition, we found no correlation between the induced T-cell proliferative responses and the HLA-class II type of individual patients (data not shown).

### T-cell responses to aggrecan and fibrillin are related to disease subtype

**Figure 3:**
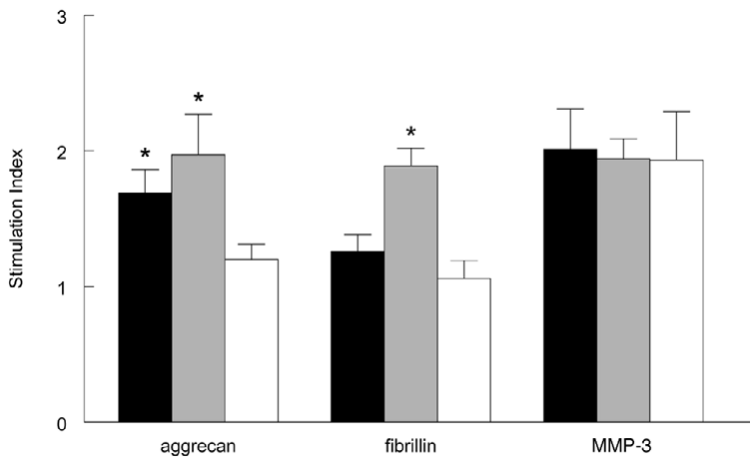
Relation of disease subtype and T-cell proliferative responses induced by aggrecan, fibrillin, and matrix metalloproteinase (MMP)-3. Proliferative responses to the aggrecan peptide of peripheral blood mononuclear cells (PBMC) from both oligoarticular and polyarticular juvenile idiopathic arthritis (JIA) patients differed significantly with those from healthy controls (*p *= 0.034 and *p *= 0.036, respectively). Only PBMC from patients with polyarticular JIA showed significant T-cell proliferative responses to the fibrillin peptide (*p *< 0.0001 versus healthy controls, *p *= 0.003 versus oligoarticular JIA). Positive proliferative responses to the MMP-3 peptide were seen in both JIA subgroups and healthy controls. Black bars: oligoarticular JIA; gray bars: polyarticular JIA; transparent bars: healthy controls. Asterisks indicate significant difference.

To investigate whether the recorded positive T-cell proliferative responses were related to JIA subtype, we split the JIA patient group into the oligoarticular and polyarticular subtypes (Figure 3). With regard to the aggrecan peptide, proliferative responses of PBMC from both oligoarticular as well as polyarticular JIA patients were significantly increased compared with healthy controls (oligoarticular JIA versus healthy controls: *p *= 0.036; polyarticular JIA versus healthy controls: *p *= 0.034). Positive T-cell proliferative responses (SI ≥ 2) were detected in 10 patients with JIA (*n *= 33): 4 patients with oligoarticular JIA (SI range 2.3 to 2.8) and 6 with polyarticular JIA (SI range 2.1 to 5.8).

In contrast, the positive proliferative responses induced by the fibrillin peptide were detected primarily in PBMC of patients with polyarticular JIA (polyarticular JIA versus healthy controls: *p *< 0.0001; polyarticular JIA versus oligoarticular JIA: *p *= 0.003). Positive T-cell proliferative responses (SI ≥ 2) were detected in 9 patients with JIA (*n *= 35): 2 patients with oligoarticular JIA (SI 2.0 in both) and 7 with polyarticular JIA (SI range 2.2 to 3.0). When comparing T-cell proliferative responses to the fibrillin peptide in oligoarticular JIA patients with those induced in PBMC of healthy controls, we could not detect a significant difference between the groups (*p *= 0.21), with low mean SI values in both groups (mean SI <1.4).

Positive T-cell proliferative responses (SI ≥ 2) to the MMP-3 peptide were detected in 16 of 36 patients with JIA (SI range 2 to 5.5) and 5 of 15 healthy controls (SI range 2 to 5.9). No significant differences in T-cell proliferative responses to the MMP-3 peptide were detected between the JIA subgroups or between the JIA subgroups and healthy controls.

### T-cell responses to aggrecan are related to disease duration

**Figure 4:**
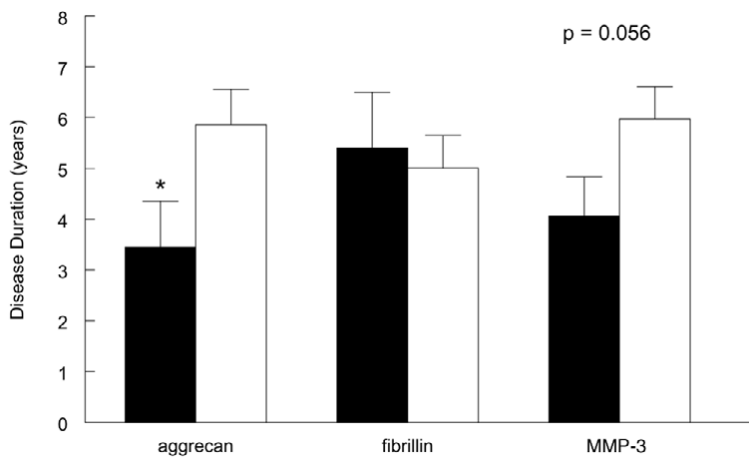
Relation of disease duration and T-cell proliferative responses induced by aggrecan, fibrillin, and matrix metalloproteinase (MMP)-3. Proliferative activity of peripheral blood mononuclear cells from patients with juvenile idiopathic arthritis was dichotomized as follows: stimulation index (SI)-non-responsive < 2 ≤ SI-responsive. Responders to the aggrecan peptide had the lowest disease duration (*p *= 0.016). Responders to the MMP-3 peptide showed a similar trend, which was close to reaching statistical significance (*p *= 0.056). Black bars: responders; transparent bars: non-responders. Asterisk indicates significant difference.

Furthermore, we analyzed whether proliferative responses to the aggrecan, fibrillin, and MMP-3 peptides were related to age, disease activity, or disease duration. When proliferative activity was dichotomized (SI-non-responsive < 2 ≤ SI-responsive), it appeared that responders to the aggrecan peptide had the lowest disease duration (*p *= 0.016) (Figure 4). With regard to the MMP-3 peptide, we found a similar trend, which was close to reaching statistical significance (*p *= 0.056). No difference in disease duration was seen between responders and non-responders to the fibrillin peptide. We could not find a relation between responders to the aggrecan, fibrillin, MMP-3 peptides and age (JIA patients or healthy controls) or disease activity (JIA patients) (data not shown).

### Peptide-specific induction of cytokines

Because the aggrecan, fibrillin, and MMP-3 peptides induced the strongest proliferative responses, these peptides were selected to analyze cytokine production of PBMC from patients with JIA and healthy controls upon peptide incubation. Using the multiplex method for cytokine analysis of supernatants taken after 96 hours of T-cell culture, we were not able to detect peptide-specific cytokine production (all mean values of IL-1α, IL-1β, IL-5, IL-10, IL-12, IL-18, TNF-α, or IFN-γ less than 2.0 pg/ml in patients with JIA and healthy controls). In addition, we performed T-cell activation assays for 72 hours and measured peptide-specific IL-4, IL-10, TNF-α, and IFN-γ production by lymphocyte intracellular staining and flow cytometry. With this technique, we did find peptide-specific induction of either cytokine, but the differences were marginal in comparison with the unstimulated PBMC and were present only in a minority of individuals (data not shown).

**Figure 5:**
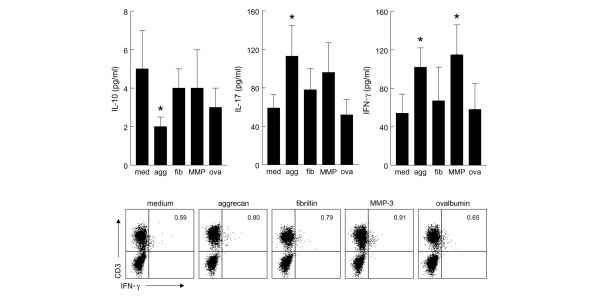
Cytokine production in short-term peptide-specific T-cell lines from patients with juvenile idiopathic arthritis (JIA). Short-term peptide-specific T-cell lines were generated from peripheral blood mononuclear cells of five patients with polyarticular JIA. After 14 days of culture, supernatants were taken for multiplex cytokine analysis and cells were stained for fluorescence-activated cell sorting (FACS) analysis. The aggrecan peptide induced significant production of interferon-γ (IFN-γ)/interleukin (IL)-17 and inhibition of IL-10 production (*p *< 0.05). The matrix metalloproteinase (MMP)-3 peptide induced significant production of IFN-γ. FACS analysis confirmed the presence of activated T cells. Asterisks indicate significant difference.

To further elaborate on potential peptide-specific cytokine production, we generated short-term peptide-specific (aggrecan, fibrillin, or MMP-3) T-cell lines of PBMC from five patients with polyarticular JIA. After 14 days of culture with repetitive peptide stimulation, supernatants were collected and cells were harvested. Analysis of supernatants revealed peptide-specific cytokine production (Figure 5). The aggrecan peptide induced the most pronounced profile of cytokines with induction of IL-17 and IFN-γ and inhibition of IL-10 production (*p *< 0.05 in all cases). In addition, the MMP-3 peptide induced significant production of IFN-γ (*p *< 0.05). Lymphocyte (intracellular) staining with CD3 and IFN-γ in combination with flow cytometry confirmed the activation of T cells in all cell lines. Representative examples are shown in Figure 5.

## Discussion

This study was conducted to identify epitopes derived from joint-related autoantigens that induce T-cell reactivity in patients with JIA. We recorded significant T-cell recognition of self-epitopes derived from aggrecan, fibrillin, and MMP-3 in patients with JIA irrespective of MHC genotype. Each of these self-epitopes shows a distinct profile of T-cell recognition in JIA and in health.

The proteoglycan aggrecan is one of the major constituents of the extracellular matrix (ECM). Degradation products of aggrecan can be detected in body fluids, including synovial fluid, where they reflect aggrecan turnover [38,39]. Previous studies show that the proteoglycan aggrecan is a target for autoreactive T cells in RA and ankylosing spondylitis [40-42]. Here, we demonstrate that aggrecan activates autoreactive T cells in JIA as well. T-cell proliferative responses to the aggrecan peptide identified were independent of JIA subtype and significantly different from those induced in healthy controls. It is well known that autoimmune disease progression is accompanied by an accumulation of neo-autoreactivity directed against target determinants not involved in disease initiation, a process known as epitope-spreading, while spontaneous regression of primary autoreactivity can occur [43,44]. We noted an inverse relation between responders to the aggrecan peptide and disease duration, which suggests that the aggrecan peptide in particular could be a target of primary autoreactivity. The induction of the pro-inflammatory cytokines IFN-γ/IL-17 and inhibition of the anti-inflammatory cytokine IL-10 indeed suggest the induction of autoaggressive T cells.

Fibrillins, also part of the ECM, are believed to guide elastogenesis and are involved in tissue homeostasis and morphogenesis. Fibrillin can be detected in the synovial lining of joints in health and disease [45]. Remarkably, T-cell proliferative responses to fibrillin are found primarily in polyarticular JIA independent of disease duration. A possible explanation for this observation may be a limited availability of fibrillin epitopes presented in oligoarticular JIA and healthy controls due to minimal or no joint destruction. To our knowledge, this is the first time that an autoantigen is described that primarily drives autoreactive T-cell responses in the more severe subtype of JIA, namely polyarticular JIA, and this autoreactivity is persistent throughout the disease course.

The MMPs are thought to be key enzymes involved in remodeling of the ECM in physiological and pathological situations. New views on the function of MMPs, however, indicate that this family of enzymes regulates various inflammatory and repair processes; matrix degradation is only one among the many functions that MMPs have [46]. MMP-3 is expressed in both JIA and normal synovial tissue, and its expression correlates with the degree of inflammation [47]. Recent studies identified MMP-3 as a target for T-cell recognition in both experimental arthritis and RA but also in age-matched healthy adult controls [27,32]. In line with these findings, we now demonstrate that the epitope derived from MMP-3 induces T-cell proliferative responses in both patients with JIA and age-matched healthy children as well. The universal recognition of the MMP-3 epitope in JIA, RA, and healthy controls (adults and children) underlines the role of MMP-3 as a target for the immune response in health and chronic arthritis. Analysis of cytokine induction via MMP-3-specific short-term T-cell lines showed primarily production of IFN-γ. This may suggest a disease-promoting role of MMP-3-specific T cells in JIA. Further studies will be necessary to determine the relevance of this finding for the pathogenesis of JIA.

Although the method of peptide selection using 'predicted' sequences in AA as a mold is slightly unusual and evidently may not yield even a semi-complete list of potential peptide sequences, the positive results support this concept. The molecular mimicry may be a critical component of T-cell responses to peptide sequences of joint-related antigens. An extensive BLAST (Basic Local Alignment Search Tool) search, however, looking for foreign peptides with an obvious sequence homology to the aggrecan, fibrillin, or MMP-3 peptide, did not yield positive results. As such, this does not support the 'molecular mimicry' hypothesis. On the other hand, it is now known that a single TCR can recognize multiple peptides that may share only one contact residue [48]. Clearly, when using this definition of molecular mimicry, our BLAST search must have been incomplete and potential mimicry epitopes must have been missed.

We analyzed T-cell responses to self-peptides derived from locally expressed non-immunodominant antigens in the joint. As such, it was not surprising that we were unable to detect significant cytokine production after direct incubation (without using any pre- or co-stimulation) of PBMC with the peptides derived from aggrecan, fibrillin, and MMP-3. The expected precursor frequency of these T cells will be low and involve low-avidity self-specific T cells [49]. This problem was overcome via generation of short-term peptide-specific T-cell lines in selected patients. Using this method, we were able to confirm the reality of the recorded T-cell proliferative responses and show that T cells indeed are activated and do secrete cytokines.

With the identification of self-epitopes that are recognized in patients with JIA, we may now use techniques like T-cell capture or tetramer staining [50-52] to sort the antigen-specific T cells directly and explore their qualities. Given that we expect the responding T cells to be low-affinity T cells, the use of tetramers will present difficulties. The T-cell capture technique, however, exploits so-called 'artificial APCs' (antigen-presenting cells) that contain high numbers of MHC-peptide complexes (signal 1) and include the presence of co-stimulatory molecules at the constructed MHC molecules (signal 2) [52]. This reflects the *in vivo *situation and indeed may allow binding of high-affinity as well as low-affinity T cells [52].

## Conclusion

This study identifies a triplet of self-epitopes that are derived from the joint-related antigens aggrecan, fibrillin, and MMP-3 that are recognized in patients with JIA. Immune reactivity to the aggrecan peptide is present in the oligoarticular and polyarticular subtypes of JIA and this peptide may be a target of primary autoreactivity. The fibrillin peptide, to our knowledge, is the first identified autoantigen specifically recognized in the polyarticular subtype of JIA. The peptide derived from MMP-3 elicits significant immune responses in chronic arthritis and in healthy controls. Further studies will be necessary to yield a more detailed view of the role of these peptides in the pathogenesis of JIA.

## Abbreviations

AA = adjuvant arthritis; BLAST = Basic Local Alignment Search Tool; cpm = counts per minute; ECM = extracellular matrix; FCS = fetal calf serum; FITC = fluorescein isothiocyanate; HLA = human leukocyte antigen; HPLC = high performance liquid chromatography; IFN-γ = interferon-γ; IL = interleukin; JIA = juvenile idiopathic arthritis; MHC = major histocompatibility complex; MMP = matrix metalloproteinase; PBMC = peripheral blood mononuclear cells; PBS = phosphate-buffered saline; PE = phycoerythrin; RA = rheumatoid arthritis; SI = stimulation index; SMPS = simultaneous multiple peptide synthesis; TCR = T-cell receptor; TNF-α = tumor necrosis factor-α.

## Competing interests

The authors declare that they have no competing interests.

## Authors' contributions

SK, KH, MK, WdJ, and MH performed all laboratory experiments. SK undertook the clinical and biostatistical analyses. MW, JvB, and SA helped with the design of the study. BP designed and supervised the study with the assistance of WK and MW. SK and BP wrote the article. All authors read and approved the final manuscript.

## Figures and Tables

**Table 1:** Characteristics of patients with JIA

	JIA	Oligoarticular JIA	Polyarticular JIA
Number	36	16 (44%)	20 (56%)
Male/Female	9/27 (25%/75%)	3/13 (19%/81%)	6/14 (30%/70%)
Age range in years (median)	3.5–18.9 (11.4)	3.5–16.6 (11.7)	4.9–18.9 (10.9)
Disease duration^a^	5.1 (3.3)	5.2 (4.0)	5.0 (2.8)
Disease activity (active/remission)	22/14 (61%/39%)	9/7 (56%/44%)	13/7 (65%/35%)
RF-positive	2 (6%)	0	2 (10%)
ANA-positive	21 (58%)	11 (69%)	10 (50%)
Uveitis	3 (8%)	2 (13%)	1 (5%)
NSAID use	30 (83%)	11 (69%)	19 (95%)
Sulfasalazine and/or methotrexate use	10 (28%)	2 (13%)	8 (40%)

^a^Expressed in years as mean (standard deviation). Disease duration at the time of sampling was defined as the time that passed since the first disease-related symptom (arthritis). The presence of joint swelling or limitation of movement with either pain on movement or tenderness defined active disease. The absence of joint swelling or limitation of movement with either pain on movement or tenderness defined disease remission. None of the patients was treated with prednisone, anti-tumor necrosis factor-α, or anti-interleukin-1 therapy. ANA, anti-nuclear antigen; JIA, juvenile idiopathic arthritis; NSAID, non-steroidal anti-inflammatory drug; RF, rheumatoid factor.

**Table 2:** Characteristics of the peptides derived from joint-related structures

Protein	Sequence	Position^a^	Abbreviation
Aggrecan core protein precursor	LSGLPSGGEVLEISV	1,352–1,366	aggrecan
Aggrecan core protein precursor	ISGLPSGGDDLETST	924–938	aggrecan3
Calpain-2	HAYSVTGAEEVESNG	262–276	calpain
Fibrillin-1 precursor	CVDTRSGNCYLDIRP	1,526–1,540	fibrillin
Matrix metalloproteinase-1	GVVSHSFPATLETQE	14–28	MMP-1
Matrix metalloproteinase-3	FFYFFTGSSQLEFDP	446–460	MMP-3
Matrix metalloproteinase-10	SAFWPSLPSGLDAAY	329–343	MMP-10
Matrix metalloproteinase-16	VKEGHSPPDDVDIVI	539–553	MMP-16
Tenascin	EPVSGSFTTALDGPS	1,789–1,803	tenascin

^a^Amino acid numbering according to the Swiss Protein Database. All peptides fulfill the computer search criteria described by van Bilsen and colleagues [25].
